# A new polymorph of 2-(2*H*-benzotriazol-2-yl)acetic acid

**DOI:** 10.1107/S1600536812036768

**Published:** 2012-09-01

**Authors:** Guloy Alieva, Jamshid Ashurov, Nasir Mukhamedov, Nusrat Parpiev

**Affiliations:** a The Mirzo Ulugbek National University of Uzbekistan, Faculty of Chemistry, University Str. 6, Tashkent 100779, Uzbekistan; b Institute of Bioorganic Chemistry, Academy of Sciences of Uzbekistan, H. Abdullaev Str. 83, Tashkent 100125, Uzbekistan; c S. Yunusov Institute of the Chemistry of Plant Substances, Academy of Sciences of Uzbekistan, Mirzo Ulugbek Str. 77, Tashkent 100170, Uzbekistan

## Abstract

A new polymorph of 2-(benzotriazol-2-yl)acetic acid, C_8_H_7_N_3_O_2_, crystallizes in the space group *C*2/*c* (*Z* = 8). The non-planar mol­ecule has a synplanar conformation of the carb­oxy group. The crystal structure features helices parallel to the *b* axis sustained by O—H⋯N hydrogen bonding which are similar to those in the known polymorph [Giordano & Zagari (1978 ▶). *J. Chem. Soc. Perkin Trans. 2*, pp. 312–315]. However, in the title structure, columns are formed by π–π stacking inter­actions between benzotriazole fragments of centrosymmetrically related adjacent mol­ecules [centroid-centroid distances = 3.593 (10) and 3.381 (10) Å] whereas π–π stacking inter­actions are not observed in the other polymorph. In the crystal of the title compound, C—H⋯O inter­actions are also observed.

## Related literature

For general background to the biological activity of benzotriazole derivatives, see: Hirokawa *et al.* (1998 ▶); Yu *et al.* (2003 ▶); Kopanska *et al.* (2004 ▶). For the previously reported polymorph, see: Giordano & Zagari (1978 ▶).
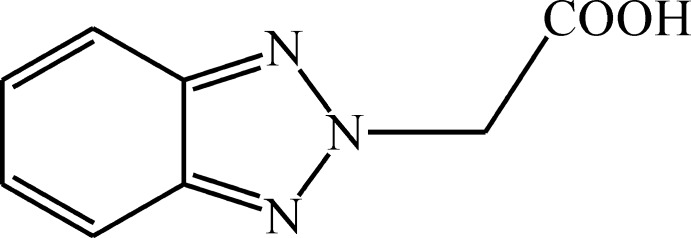


## Experimental

### 

#### Crystal data

C_8_H_7_N_3_O_2_
*M* *_r_* = 177.17
Monoclinic, 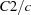

*a* = 11.719 (9) Å
*b* = 8.308 (3) Å
*c* = 17.246 (5) Å
β = 96.703 (5)°
*V* = 1667.6 (15) Å^3^
*Z* = 8
Cu *K*α radiation
μ = 0.89 mm^−1^
*T* = 293 K
0.40 × 0.32 × 0.28 mm

#### Data collection

Oxford Diffraction Xcalibur, Ruby diffractometer
Absorption correction: multi-scan (*CrysAlis PRO*; Oxford Diffraction, 2009 ▶) *T*_min_ = 0.181, *T*_max_ = 1.000
4907 measured reflections
1488 independent reflections
1235 reflections with *I* > 2σ(*I*)
*R*_int_ = 0.022

#### Refinement

*R*[*F* ^2^ > 2σ(*F* ^2^)] = 0.036
*wR*(*F* ^2^) = 0.100
*S* = 1.04
1488 reflections
120 parameters
H-atom parameters constrained
Δρ_max_ = 0.15 e Å^−3^
Δρ_min_ = −0.12 e Å^−3^

### 

Data collection: *CrysAlis PRO* (Oxford Diffraction, 2009 ▶); cell refinement: *CrysAlis PRO*; data reduction: *CrysAlis PRO*; program(s) used to solve structure: *SHELXS97* (Sheldrick, 2008 ▶); program(s) used to refine structure: *SHELXL97* (Sheldrick, 2008 ▶); molecular graphics: *XP* in *SHELXTL* (Sheldrick, 2008 ▶); software used to prepare material for publication: *SHELXL97*.

## Supplementary Material

Crystal structure: contains datablock(s) I, global. DOI: 10.1107/S1600536812036768/ds2205sup1.cif

Structure factors: contains datablock(s) I. DOI: 10.1107/S1600536812036768/ds2205Isup2.hkl

Supplementary material file. DOI: 10.1107/S1600536812036768/ds2205Isup3.cml

Additional supplementary materials:  crystallographic information; 3D view; checkCIF report

## Figures and Tables

**Table 1:** Hydrogen-bond geometry (Å, °)

*D*—H⋯*A*	*D*—H	H⋯*A*	*D*⋯*A*	*D*—H⋯*A*
O1—H1*A*⋯N3^i^	0.82	1.91	2.7273 (17)	171
C2—H2⋯O2^ii^	0.93	2.54	3.365 (3)	148
C7—H7*A*⋯O1^iii^	0.97	2.49	3.387 (3)	154
C7—H7*B*⋯O2^iv^	0.97	2.39	3.268 (3)	150

Symmetry codes: (i) 
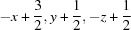
; (ii) 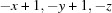; (iii) 
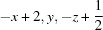
; (iv) 
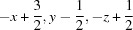
.

**Fig. 1.**
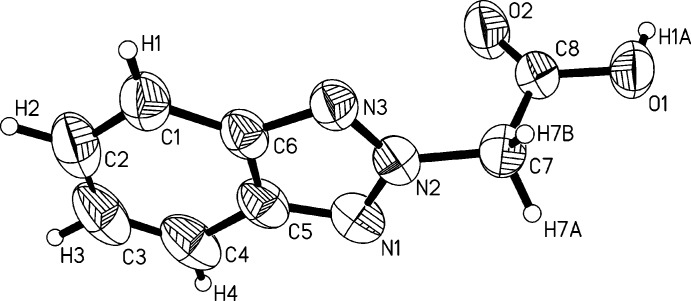
The molecular structure of (II), showing the atom-labelling scheme. Displacements ellipsoids are at the 50% probability level.

**Fig. 2.**
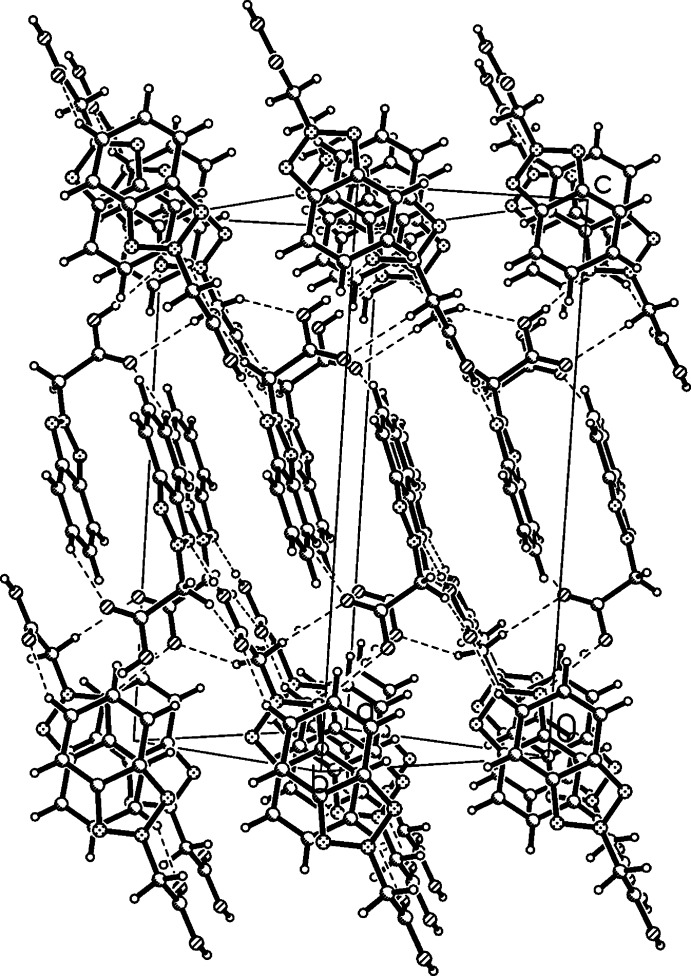
A packing diagram of (II). Hydrogen bonds are shown as dashed lines.

## supplementary crystallographic information

### Comment

Benzotriazol derivatives exhibit a good degree of analgesic, diuretic, anti-inflammatory, antiviral and antihypertensive activities (Kopanska *et al.*, 2004; Yu *et al.*, 2003; Hirokawa *et al.*, 1998).

In the known polymorphic form (polymorph I) of the title compound reported by Giordano *et al.*(1978) [J. Chem. Soc., Perkin Trans. 2, 312–315] the molecules are linked in helices parallel to *b* axis by a strong O—H···N hydrogen bond [D···A =2.6995 Å; angle D—H···A =168.08°]. We have now obtained a new polymorph of benzotriazol-2-yl-acetic acid (II), which crystallizes in the space group *C*2/*c* and its crystal structure is reported here. The asymmetric unit comprises a non-planar independent molecule with a synplanar conformation of the carboxyl group (Fig. 1). Carboxyl group is twisted away from the plane of the 1,2,3-benzotriazol fragment (C1/C2/C3/C4/C5/C6/N1/N2/N3) by 88.41 (15)°. There are helices parallel to *b* axis forming by intermolecular O—H···N hydrogen bonds [D···A =2.7273 (17) Å; D—H···A =171°] as in polymorph I (Table 1). Columns form by π-π stacking interactions between benzotriazol fragments of centrosymmetrically related adjacent molecules [centroid-centroid (1.5 - *x*, 0.5 - *y*,-*z*) distance = 3.593 (10) Å, C6···C6 (1.5 - *x*, 0.5 - *y*, -*z*) distance is 3.381 (10) Å] and [C6···C2 (1 - *x*, 1 - *y*, -*z*) distance is 3.361 (10) Å]. In the known polymorph I stacking interactions are not observed. The crystal structure of the title compound is further stabilized *via* C—H···O interactions [C2···O2 = 3.365 (3) Å; angle C2—H2···O2 = 148°, C7···O1 = 3.387 (3) Å; angle C7—H7A···O1 = 154°, C7···O2 = 3.268 (3) Å; angle C7—H7B···O2 = 150°] (Fig. 2).

### Experimental

Solid benzotriazol-2-yl-acetic acid was dissolved in dimethylformamide, &filig;ltered and left for crystallization by slow evaporation of the solvent at 30°C temperature. Colourless block crystals were obtained after two weeks.

### Refinement

H atoms were positioned geometrically, with O—H = 0.82 Å (for OH) and C—H = 0.93 and 0.97 Å for aromatic and methylene H, with *U*_iso_(H) = 1.2Ueq(C) and *U*_iso_(H) = 1.5Ueq(O).

### Crystal data

**Table:**

C_8_H_7_N_3_O_2_	*F*(000) = 736
*M**_r_* = 177.17	*D*_x_ = 1.411 Mg m^−^^3^
Monoclinic, *C*2/*c*	Cu *K*α radiation, λ = 1.54184 Å
Hall symbol: -C 2yc	Cell parameters from 408 reflections
*a* = 11.719 (9) Å	θ = 5.2–43.7°
*b* = 8.308 (3) Å	µ = 0.89 mm^−^^1^
*c* = 17.246 (5) Å	*T* = 293 K
β = 96.703 (5)°	Block, colourless
*V* = 1667.6 (15) Å^3^	0.40 × 0.32 × 0.28 mm
*Z* = 8	

### Data collection

**Table:**

Oxford Diffraction Xcalibur, Ruby diffractometer	1488 independent reflections
Radiation source: fine-focus sealed tube	1235 reflections with *I* > 2σ(*I*)
Graphite monochromator	*R*_int_ = 0.022
Detector resolution: 10.2576 pixels mm^-1^	θ_max_ = 67.1°, θ_min_ = 5.2°
ω scans	*h* = −12→13
Absorption correction: multi-scan (*CrysAlis PRO*; Oxford Diffraction, 2009)	*k* = −9→9
*T*_min_ = 0.181, *T*_max_ = 1.000	*l* = −20→19
4907 measured reflections	

### Refinement

**Table:**

Refinement on *F*^2^	Secondary atom site location: difference Fourier map
Least-squares matrix: full	Hydrogen site location: inferred from neighbouring sites
*R*[*F*^2^ > 2σ(*F*^2^)] = 0.036	H-atom parameters constrained
*wR*(*F*^2^) = 0.100	*w* = 1/[σ^2^(*F*_o_^2^) + (0.0576*P*)^2^ + 0.4124*P*] where *P* = (*F*_o_^2^ + 2*F*_c_^2^)/3
*S* = 1.04	(Δ/σ)_max_ < 0.001
1488 reflections	Δρ_max_ = 0.15 e Å^−^^3^
120 parameters	Δρ_min_ = −0.12 e Å^−^^3^
0 restraints	Extinction correction: *SHELXL97* (Sheldrick, 2008), Fc^*^=kFc[1+0.001xFc^2^λ^3^/sin(2θ)]^-1/4^
Primary atom site location: structure-invariant direct methods	Extinction coefficient: 0.0012 (3)

### Special details

**Table:**

Geometry. All e.s.d.'s (except the e.s.d. in the dihedral angle between two l.s. planes) are estimated using the full covariance matrix. The cell e.s.d.'s are taken into account individually in the estimation of e.s.d.'s in distances, angles and torsion angles; correlations between e.s.d.'s in cell parameters are only used when they are defined by crystal symmetry. An approximate (isotropic) treatment of cell e.s.d.'s is used for estimating e.s.d.'s involving l.s. planes.
Refinement. Refinement of *F*^2^ against ALL reflections. The weighted *R*-factor *wR* and goodness of fit *S* are based on *F*^2^, conventional *R*-factors *R* are based on *F*, with *F* set to zero for negative *F*^2^. The threshold expression of *F*^2^ > σ(*F*^2^) is used only for calculating *R*-factors(gt) *etc*. and is not relevant to the choice of reflections for refinement. *R*-factors based on *F*^2^ are statistically about twice as large as those based on *F*, and *R*- factors based on ALL data will be even larger.

### Fractional atomic coordinates and isotropic or equivalent isotropic displacement parameters (Å^2^)

**Table:**

	*x*	*y*	*z*	*U*_iso_*/*U*_eq_	
C1	0.55191 (14)	0.2934 (2)	−0.01273 (10)	0.0573 (4)	
H1	0.5107	0.2146	0.0103	0.069*	
C2	0.52406 (16)	0.3401 (2)	−0.08830 (10)	0.0713 (6)	
H2	0.4617	0.2915	−0.1175	0.086*	
C3	0.58612 (18)	0.4597 (3)	−0.12416 (10)	0.0763 (6)	
H3	0.5632	0.4868	−0.1760	0.092*	
C4	0.67792 (18)	0.5361 (2)	−0.08543 (9)	0.0682 (5)	
H4	0.7186	0.6142	−0.1094	0.082*	
C5	0.70856 (14)	0.49131 (18)	−0.00695 (8)	0.0511 (4)	
C6	0.64717 (12)	0.37216 (17)	0.02830 (8)	0.0454 (4)	
C7	0.85382 (12)	0.48491 (19)	0.18123 (8)	0.0517 (4)	
H7A	0.9296	0.5179	0.1701	0.062*	
H7B	0.8618	0.3840	0.2096	0.062*	
C8	0.80584 (12)	0.61084 (19)	0.23154 (8)	0.0480 (4)	
N1	0.79426 (11)	0.54651 (15)	0.04583 (7)	0.0542 (4)	
N2	0.78104 (10)	0.46083 (14)	0.10872 (7)	0.0468 (3)	
N3	0.69527 (10)	0.35514 (14)	0.10293 (6)	0.0457 (3)	
O1	0.87531 (9)	0.63393 (16)	0.29596 (6)	0.0666 (4)	
H1A	0.8481	0.7025	0.3227	0.100*	
O2	0.71669 (10)	0.67914 (16)	0.21507 (6)	0.0672 (4)	

### Atomic displacement parameters (Å^2^)

**Table:**

	*U*^11^	*U*^22^	*U*^33^	*U*^12^	*U*^13^	*U*^23^
C1	0.0544 (9)	0.0607 (10)	0.0547 (9)	0.0124 (7)	−0.0024 (7)	−0.0122 (7)
C2	0.0694 (11)	0.0844 (13)	0.0551 (10)	0.0264 (10)	−0.0136 (8)	−0.0231 (9)
C3	0.1008 (15)	0.0870 (13)	0.0384 (9)	0.0391 (12)	−0.0026 (9)	−0.0039 (9)
C4	0.0975 (13)	0.0652 (10)	0.0433 (9)	0.0255 (10)	0.0145 (9)	0.0059 (8)
C5	0.0650 (9)	0.0475 (8)	0.0414 (8)	0.0153 (7)	0.0087 (6)	−0.0012 (6)
C6	0.0507 (8)	0.0468 (8)	0.0383 (7)	0.0133 (6)	0.0031 (6)	−0.0038 (6)
C7	0.0471 (8)	0.0582 (9)	0.0482 (9)	0.0004 (7)	−0.0008 (6)	−0.0039 (7)
C8	0.0475 (8)	0.0574 (9)	0.0386 (7)	−0.0016 (7)	0.0026 (6)	0.0013 (6)
N1	0.0677 (8)	0.0487 (7)	0.0479 (7)	0.0021 (6)	0.0132 (6)	0.0007 (5)
N2	0.0512 (7)	0.0488 (7)	0.0403 (6)	0.0007 (5)	0.0046 (5)	−0.0023 (5)
N3	0.0477 (7)	0.0483 (7)	0.0403 (6)	0.0032 (5)	0.0027 (5)	−0.0008 (5)
O1	0.0524 (6)	0.0924 (9)	0.0518 (7)	0.0143 (6)	−0.0069 (5)	−0.0215 (6)
O2	0.0643 (7)	0.0858 (9)	0.0482 (6)	0.0216 (6)	−0.0072 (5)	−0.0114 (6)

### Geometric parameters (Å, º)

**Table:**

C1—C2	1.362 (3)	C6—N3	1.3510 (17)
C1—C6	1.411 (2)	C7—N2	1.4431 (18)
C1—H1	0.9300	C7—C8	1.509 (2)
C2—C3	1.415 (3)	C7—H7A	0.9700
C2—H2	0.9300	C7—H7B	0.9700
C3—C4	1.356 (3)	C8—O2	1.1937 (19)
C3—H3	0.9300	C8—O1	1.3126 (17)
C4—C5	1.409 (2)	N1—N2	1.3214 (17)
C4—H4	0.9300	N2—N3	1.3296 (17)
C5—N1	1.354 (2)	O1—H1A	0.8200
C5—C6	1.403 (2)		
			
C2—C1—C6	115.77 (18)	C5—C6—C1	121.70 (14)
C2—C1—H1	122.1	N2—C7—C8	111.85 (12)
C6—C1—H1	122.1	N2—C7—H7A	109.2
C1—C2—C3	122.69 (18)	C8—C7—H7A	109.2
C1—C2—H2	118.7	N2—C7—H7B	109.2
C3—C2—H2	118.7	C8—C7—H7B	109.2
C4—C3—C2	122.14 (17)	H7A—C7—H7B	107.9
C4—C3—H3	118.9	O2—C8—O1	124.82 (14)
C2—C3—H3	118.9	O2—C8—C7	124.56 (13)
C3—C4—C5	116.61 (18)	O1—C8—C7	110.62 (13)
C3—C4—H4	121.7	N2—N1—C5	102.73 (13)
C5—C4—H4	121.7	N1—N2—N3	117.02 (11)
N1—C5—C6	108.97 (13)	N1—N2—C7	121.50 (13)
N1—C5—C4	129.93 (17)	N3—N2—C7	121.45 (12)
C6—C5—C4	121.10 (16)	N2—N3—C6	103.30 (11)
N3—C6—C5	107.98 (13)	C8—O1—H1A	109.5
N3—C6—C1	130.32 (14)		

### Hydrogen-bond geometry (Å, º)

**Table:**

*D*—H···*A*	*D*—H	H···*A*	*D*···*A*	*D*—H···*A*
O1—H1*A*···N3^i^	0.82	1.91	2.7273 (17)	171
C2—H2···O2^ii^	0.93	2.54	3.365 (3)	148
C7—H7*A*···O1^iii^	0.97	2.49	3.387 (3)	154
C7—H7*B*···O2^iv^	0.97	2.39	3.268 (3)	150

Symmetry codes: (i) −*x*+3/2, *y*+1/2, −*z*+1/2; (ii) −*x*+1, −*y*+1, −*z*; (iii) −*x*+2, *y*, −*z*+1/2; (iv) −*x*+3/2, *y*−1/2, −*z*+1/2.
